# Muscle-Specific Dosing of OnabotulinumtoxinA in Post-Stroke Upper-Limb Spasticity: A Descriptive Literature Review

**DOI:** 10.3390/toxins18040192

**Published:** 2026-04-21

**Authors:** Małgorzata Cisowska-Adamiak, Magdalena Mackiewicz-Milewska, Elżbieta Dorota Miller

**Affiliations:** 1 Department of Rehabilitation, Faculty of Health Sciences, Collegium Medicum, Nicolaus Copernicus University, Skłodowskiej-Curie 9, 85-094 Bydgoszcz, Poland; magmami@cm.umk.pl; 2 Department of Neurological Rehabilitation, Medical University of Lodz, Milionowa 14, 93-113 Lodz, Poland

**Keywords:** botulinum toxin, onabotulinumtoxinA, post-stroke spasticity, upper limb spasticity

## Abstract

Background: Botulinum neurotoxin type A is widely used in the management of post-stroke upper-limb spasticity; however, many studies report total injected doses rather than muscle-specific dosing, limiting clinical applicability. This study aimed to evaluate how frequently muscle-level dosing protocols of onabotulinumtoxinA are reported and to assess consistency of dosing patterns across published studies. Methods: A literature search was conducted in PubMed, Wiley/Cochrane Library, and EBSCO/CINAHL using a structured search strategy informed by PRISMA guidelines. Studies published within the last 10 years reporting on onabotulinumtoxinA treatment in post-stroke upper-limb spasticity with muscle-specific dosing data were included. Studies not providing muscle-level dosing or not allowing extraction of post-stroke upper-limb data were excluded. Data were summarized descriptively and compared across studies. Results: Twenty-seven full-text articles were assessed, and five studies met the inclusion criteria. Muscle-specific dosing was consistently reported for commonly treated muscles such as biceps brachii and wrist and finger flexors, whereas other muscles were less frequently targeted. Variability in dosing between studies was observed, particularly in multicenter real-world datasets. Standardized high-dose protocols contrasted with individualized dosing strategies, which generally showed more moderate dose ranges. Expert recommendations often suggest higher doses than those observed in routine clinical practice. Conclusions: Muscle-specific dosing of onabotulinumtoxinA in post-stroke upper-limb spasticity is reported infrequently, and substantial variability exists between studies and clinical practice. Standardized reporting of muscle-level dosing and its relationship to baseline spasticity severity is needed to improve clinical applicability and reproducibility.

## 1. Introduction

Post-stroke spasticity affects approximately 20–40% of patients, depending on the time since stroke and assessment methods [1,2,3].

Botulinum toxin treatment is a well-established method for reducing focal muscle overactivity in the limbs following central nervous system injury [1,4]. Currently, the most widely used botulinum toxin type A formulations in Europe are onabotulinumtoxinA, abobotulinumtoxinA, and incobotulinumtoxinA. Botulinum toxin (BoNT) blocks acetylcholine release at the neuromuscular junction, leading to a local reduction in muscle tone [5,6]. The mechanisms underlying this process have been extensively studied and are relatively well understood at successive stages of toxin activity—from internalization into the presynaptic neuron, through translocation into the cytoplasm, to inhibition of synaptic vesicle exocytosis [7,8].

Recent evidence syntheses have confirmed the clinical effectiveness of botulinum toxin type A in the management of spasticity and highlight its widespread use across different neurological conditions [9,10]. At the same time, real-world observational studies, including large-scale programs such as ULIS, have suggested heterogeneity in treatment patterns, including muscle selection and dosing strategies [11,12,13].

As clinicians involved in this type of treatment, we are aware of the importance of precise guidance regarding dosing of a given preparation for specific muscles. However, the scientific literature demonstrates considerable variability in dosing approaches and treatment trends. The present study was developed from the need to expand knowledge regarding the practical use of a specific preparation, both in terms of administered doses and the most frequently selected muscle groups. To ensure clarity and clinical usefulness for practicing clinicians, this analysis focused on onabotulinumtoxinA.

Since botulinum toxin treatment represents a dynamically developing method for spasticity management, we aimed to analyze how frequently treatment protocols describing muscle-specific dosing are reported in the literature and to what extent these protocols demonstrate consistency and reproducibility.

To further narrow the scope of analysis and enable more precise translation of results into clinical practice and individualized patient care, this study included only the treatment of post-stroke spasticity using onabotulinumtoxinA.

Although botulinum toxin therapy is widely used in the management of post-stroke spasticity, most clinical studies report total injected doses rather than muscle-specific dosing protocols. This limits the ability to compare treatment strategies across studies and to translate research findings into clinical practice. Furthermore, it remains unclear whether dosing decisions reported in the literature take baseline spasticity severity into account.

## 2. Results

**Figure 1:**
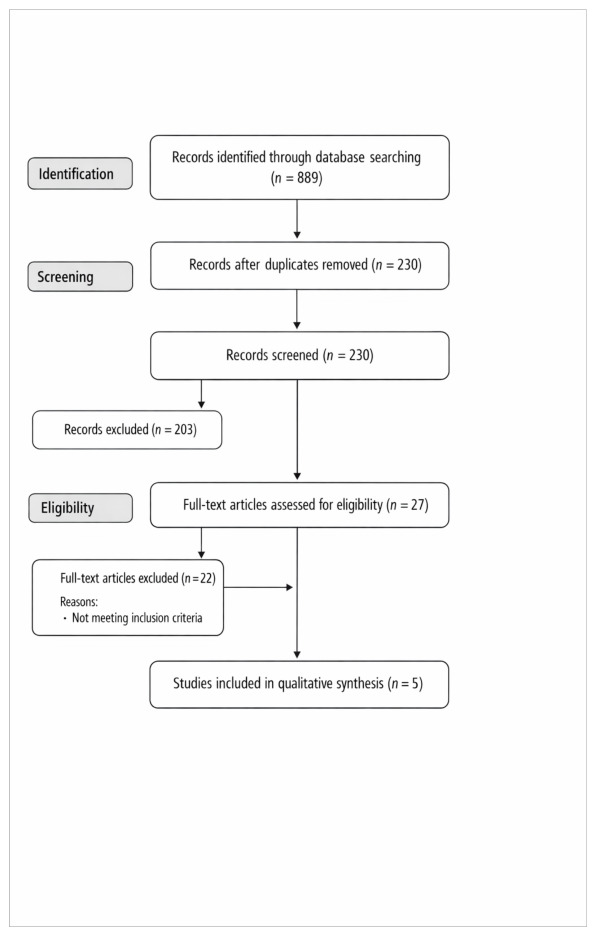
Flow diagram of the study selection process according to PRISMA guidelines. A total of 889 records were identified through database searching. After removal of duplicates, 230 records remained for title and abstract screening. Following screening, 27 full-text articles were assessed for eligibility. After applying the inclusion and exclusion criteria, 5 studies were included in the final qualitative synthesis.

A total of 889 records were identified through database searching. After removal of 659 duplicates, 230 records remained for title and abstract screening. Following screening, 27 articles were assessed for full-text eligibility. At both the abstract screening stage and full-text evaluation stage, studies were most commonly excluded for two reasons: (1) inclusion of heterogeneous spasticity populations without the possibility of extracting data exclusively for post-stroke patients, or (2) reporting botulinum toxin dosing only as total limb dose without muscle-specific breakdown. After applying inclusion and exclusion criteria, five studies were included in the final analysis. The study selection process is presented in Figure 1.

It should be noted that the study by Ranzani et al. [14] included only six participants, of whom only one patient received onabotulinumtoxinA, which significantly limits the ability to draw conclusions based on these data. The remaining publications were also based on relatively small study populations, and the largest included study involved 107 patients. The small number of eligible studies reflects the limited availability of publications reporting muscle-specific dosing data rather than restrictive inclusion criteria.

In the study conducted by Nasb et al. [15], 64 participants were included. Muscle selection for injection was performed by a clinician experienced in botulinum toxin therapy. It should be emphasized that the authors primarily focused on evaluating the clinical effects of post-stroke spasticity treatment, and the onabotulinumtoxinA injection protocol was identical for all patients. The biceps brachii muscle was injected at two sites with a dose of 200 U per site, whereas flexor carpi radialis, flexor carpi ulnaris, flexor digitorum profundus, and flexor digitorum superficialis were injected at one site with a dose of 150 U per muscle.

The largest analyzed study, conducted by Bohart et al. [16], was a retrospective multicenter report from the United States evaluating botulinum toxin dosing in upper-limb spasticity treatment. Additionally, Bohart et al. [16] conducted a survey in which 101 clinicians provided information regarding details of botulinum toxin therapy in post-stroke patients. In the analyzed group of 215 patients, 106 received onabotulinumtoxinA. A notable finding of this study was the large standard deviation values of administered doses, suggesting substantial variability in therapeutic practice between centers and physicians.

Atici et al. [17] analyzed a small group of 25 patients treated with onabotulinumtoxinA injections into spastic upper-limb muscles. The authors also evaluated changes in muscle tone and demonstrated a reduction in spasticity two weeks after treatment in shoulder adductors, elbow flexors, wrist flexors, and finger flexors. At week 12, statistically significant improvement on the Modified Ashworth Scale (MAS) was observed only for shoulder adductors. A similar trend was observed in functional assessment using the Brunnström scale—improvement was visible after two weeks but did not remain statistically significant 12 weeks after injection.

Another study included in the analysis was conducted by Ro et al. [18], which initially included 40 patients with post-stroke spasticity; however, the full treatment protocol (five botulinum toxin injections at three-month intervals) was completed by 24 patients. To improve clarity of dose presentation in the table, median doses for individual muscles were calculated based on all treatment cycles. Calculation of mean values was not possible due to a lack of muscle-specific sample size (n).

An important finding of Ro et al. [18] was a gradual increase in total dose across treatment cycles: from 272.6 ± 82.1 U during the first injection to 302.6 ± 63.8 U, 303.9 ± 67.7 U, 326.3 ± 55.3 U, and 339.1 ± 33.9 U during the second, third, fourth, and fifth cycles, respectively. The authors interpreted this as a cautious initial dosing approach due to concern about excessive muscle weakness, followed by a gradual dose increase to achieve optimal clinical effect. A tendency toward longer intervals between injections was also observed. Treatment efficacy was confirmed across all treatment cycles.

The primary aim of the study by Ranzani et al. [14] was to evaluate the effectiveness of robotic-assisted therapy combined with botulinum toxin therapy. Dose data were available for each patient; however, analysis showed that only one patient received onabotulinumtoxinA at doses presented in the table.

Across all included publications, dosing was reported for biceps brachii (BB) and wrist flexors: flexor carpi radialis (FCR) and flexor carpi ulnaris (FCU). In most studies, treatment also included brachioradialis and finger flexors, particularly flexor digitorum profundus (FDP).

Injections were reported less frequently in muscles such as pectoralis major, trapezius (horizontal portion), teres major, triceps brachii, brachialis, pronator teres, pronator quadratus, flexor pollicis longus, adductor pollicis, and lumbricals. This likely reflects the lower clinical frequency of indications for botulinum toxin injection in these muscle groups in post-stroke upper-limb spasticity.

None of the analyzed studies reported injections into latissimus dorsi, subscapularis, deltoid (middle portion), levator scapulae, or palmaris longus, nor into wrist and finger extensors such as extensor carpi radialis (ECR), extensor carpi ulnaris (ECU), extensor digitorum, or extensor pollicis brevis (Table 1).

Mean dose values with standard deviation were available in two studies (Atici and Bohart) [16,17]. In contrast, Ranzani et al. [14] reported dosing for only one post-stroke patient treated with onabotulinumtoxinA, whereas other participants received abobotulinumtoxinA. Due to heterogeneity in reporting formats (mean values, medians, and individual patient data), comparisons between studies should be interpreted with caution.

Analysis of data across studies suggests considerable variability in administered doses of onabotulinumtoxinA. However, in studies where dosing was individualized based on clinical examination, reported doses generally fell within comparable ranges. In contrast, the study by Nasb et al. [15], which applied a predefined standardized high-dose protocol, reported substantially higher doses. Among studies using individualized dosing, variability was most pronounced for the biceps brachii, for which Bohart et al. [9] reported a mean dose of 83.1 U. However, in the study by Atici et al. [17], doses were reported jointly for the biceps brachii and brachialis, limiting direct comparison between studies.

For finger flexors—flexor digitorum superficialis and flexor digitorum profundus—mean doses reported by Atici et al. [17] were approximately 40% higher than in other analyzed studies, although still substantially lower compared with the study by Nasb et al. [15], in which a dose of 150 U was used for both superficial and deep finger flexors.

A comparison between muscle-specific doses reported in clinical studies and those proposed in expert-based guidance documents is summarized in Table 2. While general agreement exists for commonly treated muscles, expert recommendations often suggest slightly higher doses than those observed in routine practice, with the exception of standardized high-dose protocols.

Because the included studies reported dosing data using different statistical measures (means, medians, or individual patient values), direct quantitative comparison between studies should be interpreted with caution. The data are therefore presented descriptively to illustrate the range and variability of reported dosing practices.

No studies provided sufficient data to allow analysis of dosing in relation to baseline spasticity severity (MAS).

## 3. Discussion

Spasticity is one of the manifestations of central nervous system damage and occurs in many neurological disorders. Each of these conditions is characterized by different clinical features, disease dynamics, and clinical determinants, which may require different therapeutic approaches using botulinum toxin. In the treatment of an individual patient, appropriate dose selection is crucial; however, it remains unclear whether dosing ranges should be identical across all disorders associated with spasticity.

However, the present analysis focuses specifically on post-stroke upper-limb spasticity, and therefore these broader considerations should be interpreted with caution.

The present review demonstrated that relatively few publications provide muscle-specific dosing data for onabotulinumtoxinA in post-stroke spasticity. This finding highlights a key gap in the literature, particularly given the widespread clinical use of botulinum toxin.

Additionally, most studies were conducted in relatively small patient populations. Nevertheless, even based on currently available data, important differences in treatment schemes using onabotulinumtoxinA can be observed. Of particular interest is the study by Nasb et al. [15], in which a predefined standardized protocol was applied for individual muscles; however, doses were sometimes substantially higher than those reported in other publications.

The remaining studies present more comparable dosing ranges. However, important observations arise from the study by Bohart et al. [16], in which very large standard deviation values were reported for most muscles, indicating substantial variability in treatment schemes. It should be emphasized that this study was based on data obtained from as many as 101 physicians. For comparison, in the study by Atici et al. [17], where injections were performed by a single clinician, standard deviations were considerably smaller despite the smaller sample size.

These findings suggest that the observed variability in dosing may be influenced by multiple factors, including injector experience, but also baseline spasticity severity, treatment goals, muscle selection strategies, and injection techniques. However, due to the limitations of the available data, these factors should be interpreted as potential contributors rather than definitive explanations.

Although expert recommendations provide general guidance on dosing, they do not consistently address key stratification factors such as baseline spasticity severity at the level of individual muscles (e.g., MAS), nor do they provide detailed guidance on muscle-specific dose adjustment in different clinical scenarios [19,20].

Comparison of data from the analyzed studies involving post-stroke patients with values presented in expert recommendations indicates that onabotulinumtoxinA dosing in clinical practice may differ substantially depending on the population, treatment center, and applied protocol. In most analyzed studies involving patients, doses for commonly injected muscles such as biceps brachii, flexor carpi radialis, and flexor carpi ulnaris were relatively similar and remained within moderate ranges. In contrast, expert recommendations often suggest higher doses (particularly for wrist flexors and pectoralis major). A clear exception is the study by Nasb et al. [15], in which a standardized treatment protocol using high doses was applied (e.g., 400 U for biceps brachii and 150 U for finger and wrist flexors), significantly exceeding values reported in other publications and in expert recommendations (Table 2).

Clinical study values are presented as reported in the original publications (mean, median, or individual doses). Expert recommendations [19,20] are based on consensus guidelines rather than primary clinical data. Observational data and expert recommendations are presented separately to reflect differences in evidence type. Abbreviations correspond to those used in Table 1.

This may suggest that, despite the availability of clinical guidelines, dose selection in spasticity treatment still largely depends on injector experience and local clinical practice. Moreover, the lack of studies analyzing dosing in relation to baseline spasticity severity (Modified Ashworth Scale, MAS) limits the development of more precise clinical dosing recommendations.

## 4. Conclusions

This review highlights important limitations in the current literature on onabotulinumtoxinA use in post-stroke upper-limb spasticity. In particular, muscle-specific dosing data are reported infrequently, and available studies show substantial variability in dosing practices across treatment centers.

Furthermore, there is a lack of studies systematically analyzing dosing in relation to baseline spasticity severity, such as that assessed using the Modified Ashworth Scale (MAS), particularly at the level of individual muscles. This limits the ability to develop precise and clinically applicable dosing recommendations.

Despite the existence of expert guidelines, considerable differences in dosing strategies are observed in clinical practice, likely reflecting heterogeneity in treatment approaches rather than standardized protocols. Clinicians should be aware of the limited availability of muscle-specific dosing data and interpret existing dosing schemes with caution, particularly in the absence of standardized guidance linked to baseline spasticity severity. These findings indicate a need for future studies providing detailed, muscle-level dosing data in clearly defined patient populations, with particular emphasis on linking dosing strategies to baseline spasticity severity.

## 5. Materials and Methods

### 5.1. Aim

This study represents a descriptive literature review with a structured search strategy aimed at mapping the available evidence regarding muscle-specific dosing of onabotulinumtoxinA.

The aim of this study was to evaluate how frequently muscle-specific dosing of onabotulinumtoxinA is reported in the literature on post-stroke upper-limb spasticity and to describe the variability of reported dosing protocols across studies. Particular attention was given to whether dosing data were reported in relation to baseline spasticity severity measured by the Modified Ashworth Scale (MAS).

The study framework was defined according to the PICO format: Population—patients with post-stroke upper-limb spasticity; Intervention—treatment with onabotulinumtoxinA; Comparison—not applicable due to the descriptive nature of the review; Outcome—reported muscle-specific dosing patterns.

### 5.2. Literature Search

The literature search was conducted in PubMed, Wiley/Cochrane Library, and EBSCO/CINAHL databases. The search covered publications from the previous 10 years and was performed in June 2025. The search was not geographically restricted.

The search strategy combined the following terms using Boolean operators: (“onabotulinumtoxinA” OR “Botox”) AND (“stroke” OR “post-stroke”) AND (“upper limb” OR “upper extremity”) AND (“dose” OR “dosing”).

### 5.3. Study Selection

Titles and abstracts were screened by a single reviewer to identify potentially eligible studies. Full texts of relevant articles were then assessed according to predefined inclusion and exclusion criteria. Data extraction was also performed by a single reviewer. This approach may introduce selection bias and is acknowledged as a limitation of the study.

### 5.4. Inclusion Criteria

Publications meeting the following criteria were included in the analysis:

- Studies describing botulinum toxin treatment in post-stroke patients;
- Studies concerning upper-limb spasticity;
- Publications containing data on muscle tone before and after treatment (if available).

If a study also included other treatment areas (e.g., lower limb) or other patient groups with different indications for botulinum toxin therapy, it was included only if data related specifically to post-stroke patients and the upper limb could be clearly extracted.

### 5.5. Exclusion Criteria

Studies were excluded if they:

- Did not provide information on doses assigned to individual muscles;
- Included patients with other neurological conditions (e.g., multiple sclerosis, cerebral palsy) and post-stroke data could not be separated.

Because the aim of the study was to present dose ranges used across different centers, and treatment efficacy was not the primary focus of the analysis, studies with small sample sizes were also included.

Only articles meeting all inclusion criteria and none of the exclusion criteria were included in the final analysis.

The 10-year time restriction was applied intentionally to reflect current clinical practice and contemporary botulinum toxin dosing strategies in spasticity treatment.

As the aim of this study was to descriptively summarize the available evidence rather than to compare treatment effects, a formal risk-of-bias assessment was not performed. This approach is consistent with the descriptive nature of the review and is acknowledged as a limitation of the study.

### 5.6. Statistical Analysis

A descriptive analysis was performed to provide a structured and transparent presentation of onabotulinumtoxinA dosing for individual upper-limb muscles in post-stroke patients. For each publication, reported doses for specific muscles were collected and summarized using measures of central tendency.

If authors reported mean values together with standard deviation (mean ± SD), these values were directly extracted. In studies where dosing was reported across multiple treatment cycles or in a format preventing calculation of a mean value (e.g., lack of muscle-specific sample size n), the median was used as the most representative measure of typical dosing. When available, minimum and maximum values (range) were also extracted to illustrate dose variability.

Due to substantial heterogeneity between included studies (differences in injection protocols, sample sizes, reporting methods, and muscle selection), meta-analysis and statistical significance testing were not performed. The aim was to describe real-world dose ranges used across centers and highlight potential differences in treatment approaches rather than compare treatment efficacy.

## Figures and Tables

**Table 1:** Muscle-specific onabotulinumtoxinA doses reported in included studies for post-stroke upper-limb spasticity.

Study	n	Data Type	PM	TH	TMJ	BB	TB	BRA	BRAD	PT	PQ	FCU	FCR	FDS	FDP	FPL	AP	Lumbricals	Notes
Nasb et al. [15]	64	Fixed protocol	–	–	–	400	–	–	–	–	–	150	150	150	150	–	–	–	predefined high-dose protocol
Bohart et al. [16]	107	Mean ± SD	–	–	–	83.1 ± 47.9	–	57.5 ± 29.9	41.7 ± 24.0	35.7 ± 22.0	32.5 ± 5.0	34.2 ± 23.8	34.9 ± 26.8	36.3 ± 16.4	37.9 ± 17.0	23.6 ± 10.5	–	–	multicentre study
Atici et al. [17]	25	Mean ± SD	40	–	–	46.54 ± 18.41 *	–	46.54 ± 18.41 *	40 ± 14.14	44 ± 5.48	–	33.3 ± 8.16	40.56 ± 10.74	60.42 ± 18.4	65 ± 7.07	–	–	–	* BB and BRA combined
Ro et al. [18]	24	Median (range)	46.25 (42.5–50.0)	–	–	52.04 (44.3–61.4)	45.0 (35.0–60.0)	–	49.3 (44.5–60.0)	–	–	35.0 (29.7–42.0)	34.84 (29.7–42.3)	34.2 (29.5–41.8)	35.3 (27.9–39.2)	18.0 (14.3–21.1)	16.7 (10.0–20.0)	20.0	repeated injections
Ranzani et al. [14]	1	Individual	–	30	50	50	–	40	50	40	–	–	20	100	–	–	–	–	single patient

Values are presented as mean ± standard deviation (SD), median (range), or individual administered doses, depending on the reporting format in each study. In studies reporting multiple treatment cycles without muscle-specific sample sizes, median values across cycles were calculated. Direct comparison between studies should be interpreted with caution due to heterogeneity in reporting formats. Abbreviations: PM, pectoralis major; TH, trapezius (horizontal part); TMJ, teres major; BB, biceps brachii; TB, triceps brachii; BRA, brachialis; BRAD, brachioradialis; PT, pronator teres; PQ, pronator quadratus; FCU, flexor carpi ulnaris; FCR, flexor carpi radialis; FDS, flexor digitorum superficialis; FDP, flexor digitorum profundus; FPL, flexor pollicis longus; AP, adductor pollicis. – Indicates that no dose of botulinum was injected. * Dose reported jointly for biceps brachii (BB) and brachialis (BRA).

**Table 2:** Comparison of muscle-specific onabotulinumtoxinA doses reported in clinical studies and expert-based recommendations.

Study/Source	n	Data Type	PM	BB	BRA	BRAD	PT	PQ	FCU	FCR	FDS	FDP	FPL	AP	Lumbricals
**Observational studies**															
Nasb et al. [15]	64	Fixed protocol	–	400	–	–	–	–	150	150	150	150	–	–	–
Bohart et al. [16]	107	Mean ± SD	–	83.1	57.5	41.7	35.7	32.5	34.2	34.9	36.3	37.9	23.6	–	–
Atici et al. [17]	25	Mean ± SD	40	46.54 *	46.54 *	40	44	–	33.3	40.56	60.42	65	–	–	–
Ro et al. [18]	24	Median	46.25	52.04	–	49.3	–	–	35.0	34.84	34.2	35.3	18.0	16.7	20
Ranzani et al. [14]	1	Individual	–	50	40	50	40	–	–	–	20	100	–	–	–
**Expert recommendations**															
Simpson et al. [19]	–	Consensus	75	50	–	75	25	–	50	50	50	50	40	–	–
Sandrini et al. [20]	–	Consensus	75	50	35	70	50	40	30	50	55	55	35	20	35

- Indicates that no dose of botulinum was injected. * Dose reported jointly for biceps brachii (BB) and brachialis (BRA).

## Data Availability

No new data were created or analyzed in this study.

## Abbreviations

The following abbreviations are used in this manuscript:

BoNT/A	Botulinum neurotoxin type A
MAS	Modified Ashworth Scale
BB	Biceps brachii
FCR	Flexor carpi radialis
FCU	Flexor carpi ulnaris
FDP	Flexor digitorum profundus
FDS	Flexor digitorum superficialis
ECR	Extensor carpi radialis
ECU	Extensor carpi ulnaris
TH	Trapesius horyzontalis
SD	Standard deviation
